# Strategies for identifying stable lentil cultivars (*Lens culinaris* Medik) for combating hidden hunger, malnourishment, and climate variability

**DOI:** 10.3389/fpls.2023.1102879

**Published:** 2023-07-13

**Authors:** Muraleedhar S. Aski, Gyan Prakash Mishra, Jayanti P. Tokkas, Prachi S. Yadav, Neha Rai, Ruchi Bansal, Akanksha Singh, Sanjeev Gupta, Jitendra Kumar, Ashok Parihar, Shiv Kumar, Vinod Kumar, Ashok Kumar Saxsena, Tapas Ranjan Das, Anil Kumar, Harsh Kumar Dikshit

**Affiliations:** ^1^ Division of Genetics, Indian Council of Agricultural Research (ICAR)-Indian Agricultural Research Institute, New Delhi, India; ^2^ Division of Biochemistry, Chaudhary Charan Singh (CCS), Hissar Agricultural University, Hissar, Haryana, India; ^3^ Division of Plant Physiology, Indian Council of Agricultural Research (ICAR)-Indian Agricultural Research Institute, New Delhi, India; ^4^ Amity Institute of Organic Agriculture, Amity University, Noida, India; ^5^ Krishi Bhavan, Indian Council of Agricultural Research (ICAR), Delhi, India; ^6^ Division of Crop Improvement, Indian Council of Agricultural Research (ICAR) – Indian Institute of Pulses Research, Kanpur, UP, India; ^7^ Regional Research Station, International Center for Agricultural Research in the Dry Areas (ICARDA), New Delhi, India; ^8^ Regional Agricultural Research Station, Jawaharlal Nehru Krishi Vishwavidyalaya (JNKVV), Sagar, Madhya Pradesh, India; ^9^ RVSKVV-Rajmata Vijayaraje Scindia Krishi Vishwa Vidyalaya, Department of Plant Breeding & Genetics, College of Agriculture, Sehore, MP, India; ^10^ Indian Council of Agricultural Research (ICAR)-Indian Agricultural Research Institute (IARI) Regional Research Station, Pusa Samastipure, Bihar, India; ^11^ Department of Plant Breeding & Genetics, Bihar Agricultural College, Bihar Agriculture University, Bhagalpur, Bihar, India

**Keywords:** micro-nutrients, hidden hunger, anti-nutrients, stability, bio-fortification, lentil grain iron, grain zinc, phytic acid

## Abstract

Iron and zinc malnutrition is a global humanitarian concern that mostly affects newborns, children, and women in low- and middle-income countries where plant-based diets are regularly consumed. This kind of malnutrition has the potential to result in a number of immediate and long-term implications, including stunted growth, an elevated risk of infectious diseases, and poor development, all of which may ultimately cause children to not develop to the fullest extent possible. A determination of the contributions from genotype, environment, and genotype by environment interactions is necessary for the production of nutrient-dense lentil varieties that offer greater availability of iron and zinc with a high level of trait stability. Understanding the genotype and environmental parameters that affect G x E (Genotype x Environment) interactions is essential for plant breeding. We used GGE(Genotype, Genotype x Environment interactions) and AMMI (Additive Main effects and Multiplicative Interaction) models to study genetic stability and GE(Genotype x Environment interactions) for grain Fe, Zn, Al, and anti-nutritional factors like phytic acid content in sixteen commercially produced lentil cultivars over several different six geographical locations across India. Significant genetic variability was evident in the Fe and Zn levels of different genotypes of lentils. The amounts of grain iron, zinc, and phytic acid varied from 114.10 to 49.90 mg/kg, 74.62 to 21.90 mg/kg, and 0.76 to 2.84 g/100g (dw) respectively. The environment and GE (Genotype x Environment interactions) had an impact on the concentration of grain Fe, Zn, and phytic acid (PA). Heritability estimations ranged from low to high (53.18% to 99.48%). The study indicated strong correlation between the contents of Fe and Zn, a strategy for simultaneously increasing Fe and Zn in lentils may be recommended. In addition, our research revealed that the stable and ideal lentil varieties L4076 (Pusa Shivalik) for Fe concentration and L4717 (Pusa Ageti) for Zn content, which have lower phytic acid contents, will not only play an essential role as stable donors in the lentil bio-fortification but will also enable the expansion of the growing area of bio-fortified crops for the security of health and nutrition.

## Introduction

Micronutrient malnutrition, which affects more than a quarter of the world’s population, is a serious global health issue. (Gonmei and Toteja, 2018). The most typical cause of anemia, affecting 27% of the global population (Ning and Zeller, 2019), is iron deficiency. Symptoms of anemia and iron deficiency include mental impairment, lowered immunity, fatigue, early birth in neonates, and higher rates of morbidity and mortality (Cappellini et al., 2019; Jamnok et al., 2020). The oxygen-carrying proteins myoglobin and hemoglobin need iron to function (Fava et al., 2019). According to a study (Lynch et al., 1984), the amount of Fe consumed from diets based on legumes ranges from 0.8 to 1.9 percent.

According to scientific forecasts, between 17.6% and 29.6% of the world’s population will have poor zinc consumption, with Sub-Saharan Africa, South and Southeast Asia, and Central America showing the largest prevalences (Gupta et al., 2020a). Infections brought on by insufficient zinc intake have been linked to a major part of child mortality. In biological processes, zinc functions as a catalyst, structural ion, and regulatory ion (Grüngreiff et al., 2020). Due to its involvement in several metabolic processes, zinc is necessary for healthy body growth and development. Zinc insufficiency results in immune system issues, epidermal issues, hypogonadism, problems with the central nervous system, and growth retardation. (Chasapis et al., 2020). The only way to correct a zinc deficiency is through continuous zinc intake because the human body cannot store zinc. Phytic acid (PA) serves as the most potent absorption inhibitor (Sandberg, 2002). Phytate has been shown to decrease Ca, Zn, and Fe absorption in humans in a dose-dependent way (Fredlund et al., 2002). The reduction of Fe and Zn absorption by inositol pentaphosphate has also been demonstrated (Sandberg et al., 1999).The National Institute of Health (NIH) specifies that the Recommended Dietary Allowances (RDAs) for iron is 8 mg for men and 18 mg for women, but the RDA for zinc is 11 mg and 8 mg for men and women, correspondingly. The grain legume lentil (*Lens culinaris* Medikus. *culinaris*) is rich in macro- and micronutrients as well as protein, vitamins, and prebiotic carbohydrates (Taleb et al., 2013; Gupta et al., 2018; Khazaei et al., 2019). Daily consumption of 100 grams of lentils can supply significant amounts of Zn and Fe (Thavarajah et al., 2009; Kumar et al., 2018). In many poor nations, lentils are supplemented to cereals in low-income people’s daily meals (Ozer et al., 2010). Lentil is a promising grain for micronutrient biofortification since it is high in Zn and Fe and may be grown in areas where people are malnourished and face economic issues. (Singh et al., 2017a; Singh et al., 2017b).

Biofortification, a traditional or molecular breeding-based method, can boost the nutritional content of food crops by enhancing bioavailability (Garg et al., 2018). Significant genetic diversity in the genetic pool for the desired characteristic is necessary for breeding micronutrient-rich crops. It has been demonstrated that the levels of Fe and Zn in lentil germplasm vary significantly (Thavarajah et al., 2011; Karakoy et al., 2012 and Sen Gupta et al., 2013). At Indian and international level breeding efforts are made to identify genetic variability and stability in grain minerals in food grain legumes like chickpea (Erdemci, 2018; Misra et al., 2020), mungbean (Ullah et al., 2011), lentil (Darai et al., 2020), soybean (Mwiinga, 2018), faba bean (Fikere et al., 2008; Tekalign et al., 2017). In addition, IARI New Delhi delivered its first iron-rich lentil variety, Pusa Agethi Masoor, while IIPR Kanpur presented IPL 220 for commercial cultivation to farming community (Yadava et al., 2020).

Its heredity is complex since the environment heavily regulates grain micronutrient concentration (Kumar et al., 2018). The GGE model aids in identifying winning genotypes suitable for various environments and ranking them in tested environments in terms of performance, albeit the AMMI model aids in understanding the structure of GEI (Genotype Environment Interaction), trying to predict the total deviation of interaction, and distinguishes the main interactions from each other (Ebdon and Gauch, 2022a; Ebdon and Gauch, 2022b). Genotypes with stable micronutrient concentration performance across conditions can be exploited when breeding bio-fortified lentil cultivars.

This study focused on the genotype by environment interaction (G x E) across various environments in order to identify stable Fe and Zn-rich genotypes with lower phytic acid concentration.

## Material and methods

### Plant material and field experiment

In the present study, 16 commercially available lentil cultivars generated in different lentil breeding facilities in India were utilized (**Table 1**). These 16 cultivars were grown in six distinct geographical locations; each location is representative of a unique lentil growing zone, officially demarked by the All India Co-ordinated Research Project (AICRP) on MULLaRP (Mungbean, Uradbean, Lentil, Lathyrus, and Pea (field) in India.: i) ICAR-IARI New Delhi (North-West Plain Zone); (ii) ICAR-IIPR Kanpur, Uttar Pradesh (UP) (North-East Plain Zone); (iii) Sehore, Madhya Pradesh (MP) (Central Zone); (iv) Sabour, Bihar (BR) (North-East Plain Zone); (v) Samastipure, Bihar (BR) (North-East Plain Zone); and (vi) Sagar Madhya Pradesh (MP) (Central Zone) (**Table 2**).

**Table 1:** Information regarding the lentil genotypes used in the study.

S.No	Genotype	Pedigree	Developing center	Average yield(Q/ha)	Days to maturity	Reaction to Major diseases
1	DPL 62 (Sheri)	JLS-1 x LG 171	ICAR-Indian Institute of Pulses Research, Kanpur, India	17-18	130-135	Resistant to rust and tolerance wilt
2	L 4596 (Pusa Masoor-6)	LC 68-17-3-5 x L 4602	ICAR-Indian Agricultural Research Institute, New Delhi, India	20-22	120-124	Resistant to rust
3	IPL 321	DPL-62 x K 75	ICAR-Indian Institute of Pulses Research, Kanpur, India	9-10	130-135	Resistant to rust and wilt
4	L4147(Pusa Vaibhav)	(L 3875 x P4) x PKVL	ICAR-Indian Agricultural Research Institute, New Delhi, India	17-18	130-135	Resistant to rust
5	DPL 58	PL 639 X PRECOZ	ICAR-Indian Institute of Pulses Research, Kanpur, UP, India	15-18	130-135	Resistant to wilt
6	JL 3	Land race selection from Sagar MP	Jawaharlal Nehru Krishi Vishwa Vidyalaya (JNKVV) Sehore, MP, India	14-15	110-115	Resistant to wilt
7	WBL 77	ILL7723 x BLX88176	Bidhan Chandra Krishi Vidyalaya (BCKV), Berhampore, WB, India	14-15	115-120	Resistant to rust
8	L 4076	PL 234 x PL 639	ICAR-Indian Agricultural Research Institute, New Delhi, India	14-15	135-140	Resistant to rust
9	BM 4	ILL 5888 x ILL 5782	ICARDA Syria for BARI Bangladesh	20-23	116-120	Resistance to lentil rust and Stemphylium blight
10	K 75 (Malika)	Selection from Bundelkhand region	C.S. Ajad University of Agriculture and Technology, Kanpur, India	13-14	130-135	–
11	VL 520	DPL 15 x SEHORE 74-3	ICAR-Vivekananda Parvatiya Krishi Anusandhan Shansthan, Almora, India	14-15	118-120	Resistant to rust
12	PL7	L 4076 x DPL 15	G.B. Pant University of Agriculture and Technology, Pantnagar, India	16-18	125-145	Resistant to rust
13	PL 6	Pant L 4 x DPL 55	G.B. Pant University of Agriculture and Technology, Pantnagar, India	16-18	125-145	Resistant to rust
14	L 4717	ILL 7617 x 91516	ICAR-Indian Agricultural Research Institute, New Delhi, India	12-13	96-106	Resistant to wilt and AB
15	PL 406(IPL 406)	DPL 35 x EC 157634/382	G.B. Pant University of Agriculture and Technology, Pantnagar, India	13-14	120-155	Resistant to rust and wilt
16	PL 639	L 9-12 x T 8	G.B. Pant University of Agriculture and Technology, Pantnagar, India	20-22	140-150	Resistant to rust

**Table 2:** Descriptions regarding test locations in India (2018-2019) during Rabi (Oct-March).

S.No	Location Details	Biplot Name	Elevation (msl)	Latitude and longitude	Total Rain in season (mm)	RH (%)	Temp(O^C^)		
							Max	Mini	Mean
1	ICAR- Indian Agricultural Research Institute (IARI) New Delhi	Delhi	235	28.65401 77.17172	48.32	77.36	25.87	14.89	20.38
2	ICAR- Indian Institute of Pulses Research (IIPR) Kanpur, Uttar Pradesh	Kanpur	130	26.49222 80.27682	49.18	75.66	25.38	15.26	20.32
3	Jawaharlal Nehru Krishi Vishwa Vidyalaya (JNKVV) Regional Agricultural Research Station (RARS), Sagar, Madhya Pradesh(MP)	Sagar	542	23.84083 78.74582	39.57	69.89	32.11	15.85	23.98
4	Rajmata Vijayaraje Scindia Krishi Vishwavidyalaya (RKVV) Sehore, Madhya Pradesh(MP)	Sehore	502	23.21235 77.08011	40.35	70.49	31.21	18.35	24.78
5	IARI-Regional Research Station(RAS) Samstipore, Bihar	Samstipore	47	25.84522 85.78377	42.41	79.65	31.47	15.60	23.53
6	Bihar Agricultural University (BAU) Sabour, Bihar	Sabour	87	32.803056 74.061389	51.1	78.42	30.52	14.30	22.41

The soil’s properties, including pH, EC, organic carbon (OC), accessible nitrogen, phosphorus (P), and potassium (K), as well as soil texture, are presented in **Supplementary Table 1**. In three regions (Delhi, Kanpur, and Sehore), mungbean was previously planted; in the other three, blackgram was cultivated. No basal fertilization or micronutrient spraying has taken place. DAP (Di Ammonium Phosphate) was the only fertilizer used, and it was applied at a rate of 100 kg/ha. To allow adequate uniformity, the topsoil was carefully shredded and mixed, and the land was leveled in each location. The plants were planted in a randomized block design (RBD) with a plant to plant spacing of 5 cm, a row to row distance of 30 cm, and a row length of 5 m, for a total of three repetitions per entry (6 rows each replication). Crop cultivation was carried out using standard agronomic methods. Employing recognized techniques, the amounts of Fe and Zn in soil were determined (Singh et al., 2005).

### Estimation of seed iron (Fe), zinc (Zn) and aluminum (Al) concentration

Physiologically matured seeds were plucked and dried in the shade. The seeds are given two ethanol rinses to remove dust particles. To avoid metal and dust contamination, 10 g of seeds from each entry were ground into a fine powder (approximately average diameter size of 10 microns (10^-3^ cm)) using a mortar and a pestle. The microwave

digestion apparatus (Anton Parr: Multiwave ECO) was used to process the 0.5 g sample of ground grain powder in line with the modified di-acid technique (Singh et al., 2005). Fe, Zn, and Al concentrations (in ppm) were measured using self-sampling techniques with inductively coupled plasma mass spectrometry (ICP-MS) (, Model: NexION 300, ICP-MS, manufactured in USA by Perkin Elmer inc.) Aluminum (Al) was measured at 167.000 nm, Fe at 238.204nm and Zn at 213.856 nm. ICP-MS has the lower detection limit can extend to parts per trillion (ppt), while the linear range of ICP-MS is 10-11 orders of magnitude. The kits for organic solvents used in as fallows Nitric acid (HNO_3_) and hydrochloric acid (HCl) Sigma Aldrich, Merck KGaA, Darmstadt, Germany) were purified in perfluoralkoxy-polymer (PFA) sub-boiling units (DST-4000, Savillex corporation, Eden Prairie, MN 55344-3446 USA). Hydrogen peroxide solution (H_2_O_2_) Merck KGaA, Darmstadt, Germany) and tetrafluoroboric acid (HBF4) Sigma Aldrich, Merck KGaA, Darmstadt, Germany). Aluminium (Al) was identified as an indicator element in global research efforts. Fe and Zn quantification was not performed on samples that had an Al concentration of more than 5 ppm. These samples were re-washed with 70% ethanol to remove any dust contamination before being reanalyzed. Aluminum (Al) worked as an indicator element for possible potential dust contamination in this investigation as per HarvestPlus guidelines (Pfeiffer and McClafferty, 2007).

### Determination of seed phytic acid (PA) content

Phytic acid (PA) was estimated using the K-PHYTA (Megazyme International) standard assay methodology as the phosphorus produced by phytase and alkaline. Inositol phosphates are used to extract the acid, which is then processed using phytases that are specific to phytic acid (IP6) and lower myo-inositol phosphate types (i.e. IP2, IP3, IP4, IP5). Alkaline phosphatase treatment causes the final phosphate, which is very resistant to phytase activity, to be released from myo-inositol phosphate (IP1). The total amount of phosphate released is calculated and expressed in grams of phosphorus per 100 g sample using a modified colorimetric method. A calibration curve with predetermined phosphorus content standards is used to convert Pi to phosphorus. Standard phosphorus concentration curve, Standard curve: y = 0.00461 + 0.16857x Linearity: R 2 = 0.99. Concentration range: standard assay procedure this corresponds to a phosphorus concentration of ~ 2.82 mg to ~ 11.29 mg/100 g (or phytic acid concentration of ~ 10 mg to ~ 40 mg/100 g). Finally, the percentage of phytic acid is computed on the presumptions that phytic acid accounts for all of the observed phosphorus and that 28.2% of phytic acid is present (Singh et al., 2017a).


Phytic acid (g/100g)=phosphorus(g/100g)/0.282

### Construction of GGE biplot

The GGE biplot was designed depending on the first two principal components (PCs) produced *via* singular value decomposition (SVD) after computing each component of the matrix using the suggested equation. (Yan et al., 2000; Yan and Falk, 2002, and Yan and Kang, 2022). The model used is given in Eq 1:


$${\text{Y}}_{\text{ij}}=\text{μ}+{\text{e}}_{\text{j}}+\sum_{\left(\text{n}-1\right)}^{\text{N}}{\text{λ}}_{\text{n}}{\text{γ}}_{\text{in}}\;{\text{δ}}_{\text{jn}}+{\text{ϵ}}_{\text{ij}}$$

Where,

Y_ij_ = mean response of i^th^ genotype (i = 1,…,I) in the j^th^

environment (j = 1,.,J).

μ = grand mean.

e_j_ = environment deviations from the grand mean.

**λ**_n_ = the eigen value of PC analysis axis.

γ_in_ & **δ**_jn_ = genotype and environment PCs scores for axis n.

N = number of PCs retained in the model.

ϵ_ij_ = residual effect_ N (0,s2).

A “average environment coordination” (AEC) viewpoint of the GGE biplot has been constructed for genotype evaluation and stability determination, enabling genotype comparisons based on mean values of grain minerals (iron, zinc), phytic acid content, and stability between locations within a “mega-environment.” (Yan, 2001; Yan and Rajcan, 2002). The average performance of the genotype was determined using a performance line that passed through the origin of the biplot. The performance line’s arrow indicates a decline in genotype stability (Yan and Falk, 2002). The “ideal” test environment should be both genetically discriminating and representative of the “mega-environment,” according to the “discriminating power vs. representativeness” viewpoint of the GGE biplot, which was developed for the evaluation of test environments (Yan et al., 2007). The “repeatability” of a test environment was assessed using the average rating of the genetic correlations across years within the settings for sustaining stability in genotypic performances (Yan et al., 2011). The AEC has also been used to create a “desirability index” for the test sites that considers the relationship between environmental factors and ideal genotype lengths as well as genotypic stability and adaptability (Yan and Holland, 2010). To evaluate the relationship between test sites and surrounding environments, angles within different location vectors were used (Yan and Kang, 2002). In order to determine genotype dominance across several testing scenarios and to combine testing environments into separate “mega environments,” a “which won-where” GGE biplot viewpoint was also developed (Yan and Rajcan, 2002). Bootstrapping, a nonparametric re-sampling approach, was used to construct CL at the 95 percent level for each principal component value of both genotypes and environments in order to assess the validity of the GGE biplot (Yang et al., 2009).

### Data analysis

Analysis of variance (ANOVA) was used to evaluate the effects of environments, genotypes, and their interactions across sites and for each individual genotype using mixed model analysis in R software. A combined analysis of variance (ANOVA) across locations was carried out after an error variance homogeneity test based on Bartlett’s test. Stability was investigated using the AMMI and genotype + genotype x environment (GGE) models. The AMMI1 biplot was plotted using the mean of the main effect vs. the first interaction principal component (IPC1) score (Zobel et al., 1988). The ANOVA demonstrated how the variance distribution was impacted by genotypes, environment, and their interaction. The LSD test was used to calculate the mean significant difference between genotypes and environments at the P = 0.05 level of probability. A box plot was used to show how the mineral (Fe & Zn) and PA content varied among genotypes and locations. The Ward method was utilized to establish the hierarchical cluster that represented the genetic and environmental relatedness. Using R software (R Core Team, Vienna, Austria, https://www.R-project.org), the GGE biplot analysis was performed out.

## Results

### Analysis of variance

**Figure 1:**
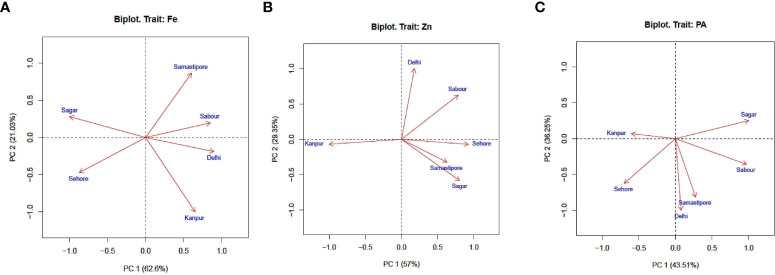
Principal component analysis (PCA) illustrating significant difference among test environments and for **(A)** Fe, **(B)** Zn, and **(C)** PA.

The study finds that several lentil genotypes responded differently with seed iron, zinc, and phytic acid. The pooled ANOVA showed that the genotypes under investigation were significantly influenced by genotype, environment, and genotype x environment interactions. For seed iron and zinc, the genotype and environment interaction produced a high estimate of the sum of squares (SS). On the other hand, phytic acid (PA) revealed more about genotype. The relative contribution of each source of variation to the total variation was estimated for seed iron (29.30%), zinc (40.99%), environment (61.85%), and GEI (18.06%) (**Table 3**). This showed an unexpected environmental influence on the mineral content of seed among genotypes tested in diverse locations. For seed iron, zinc, and phytic acid, genotype and genotype x environment interactions were significant across all genotypes examined at the various testing sites. Environmental variations showed that the habitats were unique, and they may explain a sizable portion of the variation in Fe, Zn, and PA. The illustrations for the biplot analysis were produced using these data. Biplot analysis was carried out and presented by plots to make distinctions between these environments, to assess stable and wide adaptive lines, and to assess the environments to determine whether a particular graph depicts the ideal environment to choose genotypes based on these parameters. Genotype Environment Interaction (GEI) was clearly evident in the AMMI 1 model when the interaction was divided among the first three Interaction Principal Component Axis (IPCA). Each and every PCA had statistically significant results (PCA 1, PCA 2, and PCA 3). Grain Fe, PC1 is responsible for 62.6 of the total variation. PC2 is in responsibility of 21.03 percent of the total variation, while IPC3 is in charge of 6.01 percent of the variation and has a Pr. F value above 0.005. PC1 and PC2 may be responsible for 83.63 percent of the variance in the Fe study. While PC1 and PC2 accounted for 86.35 and 79.76 percent, respectively, of the variation in Zn and PA (**Figure 1**).

**Table 3:** Analysis of variance for seed iron, zinc and phytic acid content in lentil cultivars evaluated at six locations in India during (2018–2019).

Source of variation	Degrees of freedom DF	Seed Iron (Fe)			Seed Zinc (Zn)			Phytic Acid (PA)		
		SS	MSS	%TSS	SS	MSS	%TSS	SS	MSS	%TSS
ENV	5	13589.15	2717.83***(0.000)	29.30	7296.073	1459.21***(0.000)	20.10	4.17	0.83***(0.000)	3.74
GEN	15	13774.91	918.33***(0.000)	29.70	6556.436	437.10***(0.000)	18.06	99.63	6.64***(0.000)	93.00
GEN *ENV	75	19008.78	253.45***(0.000)	40.99	22454.46	299.39***(0.000)	61.85	7.81	0.10***(0.000)	100.00
PC1	19	7581.14	399.01***(0.000)	39.88	10254.75	539.72***(0.000)	45.67	3.17	0.17***(0.000)	40.52
PC2	17	4951.10	291.24***(0.000)	26.05	6535.867	384.46***(0.000)	29.11	2.36	0.14***(0.000)	70.69
PC3	15	4080.40	272.03***(0.000)	21.47	2697.802	179.85***(0.000)	12.01	1.81	0.12***(0.000)	93.81
PC4	13	1536.91	118.22***(0.000)	8.09	2107.734	162.13***(0.000)	9.39	0.33	0.03* (0.245)	98.00
PC5	11	859.22	78.11***(0.000)	4.52	858.3005	78.03** (0.007)	3.82	0.16	0.01*(0.275)	100.00
PC6	9	0.0	0.00 (1.00)	0.00	0	0.00 (1.00)	0.00	0.00	0.00 (1.00)	100.00
Residuals	192	1616.75	8.42	0.00	6669.495	34.74	0.00	4.68	0.02	0.00

* Significant at P ≤ 0.05 respectively; ** Significant at P ≤ 0.01 respectively; *** Significant at P ≤ 0.001respectively.

**Figure 2:**
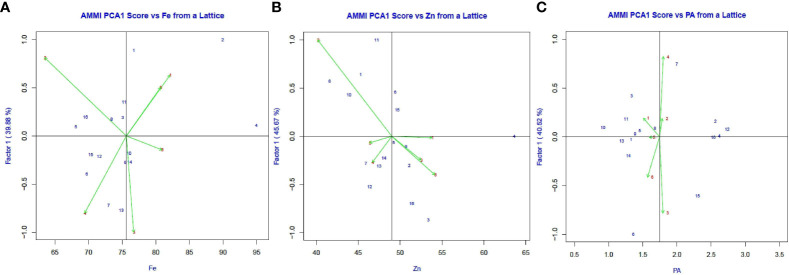
The AMMI1 Biplots (Means vs PC1) the first principal component (PC1) and mean values for **(A)** Fe, **(B)** Zn, and **(C)** PA in six environments.

**Figure 3:**
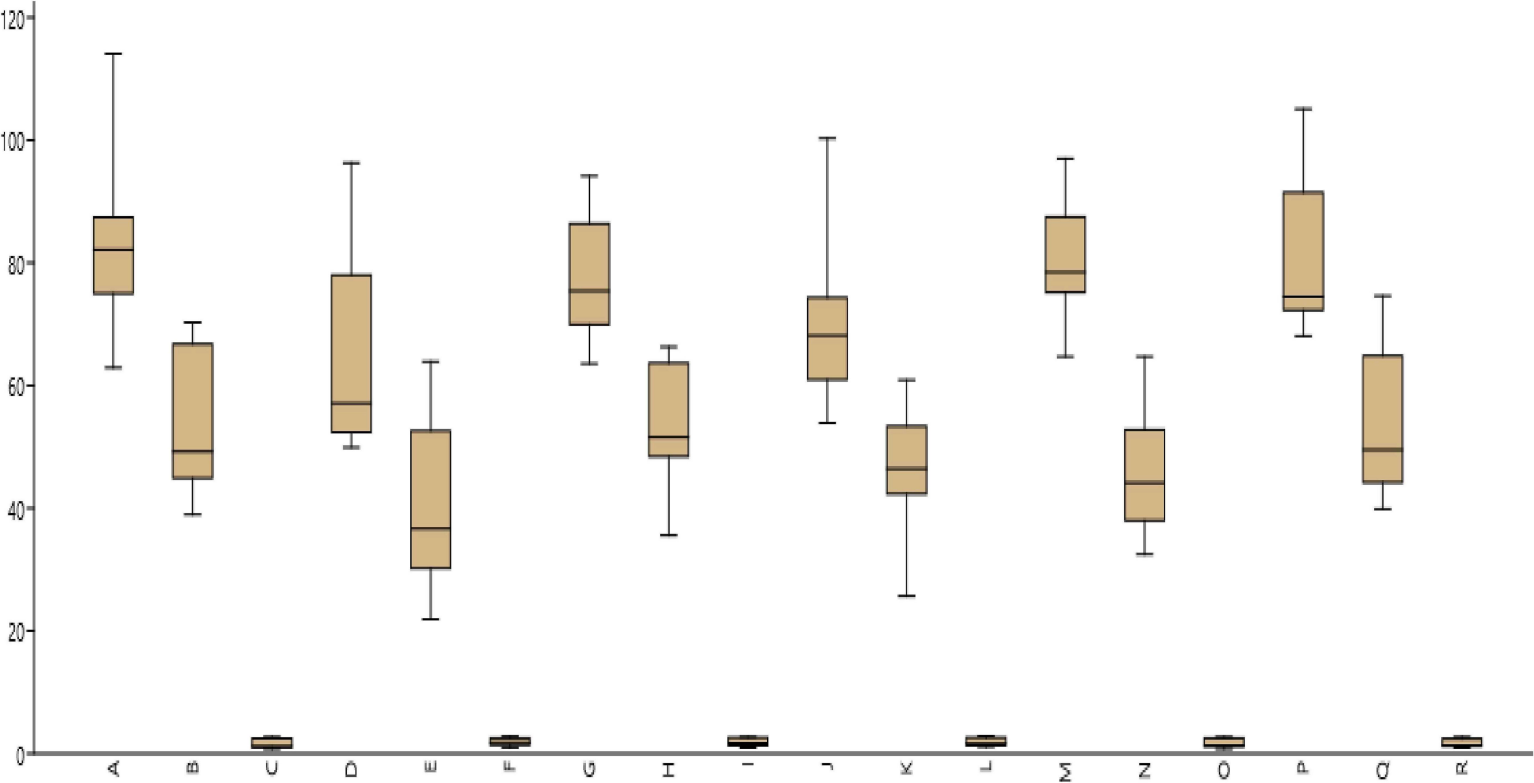
Box plot mean illustrating significant difference among test environments and for Fe, Zn and PA.

The AMMI1 Biplots (Means vs PC1) indicate the genotypes DPL 62, L 4596, and L 4147 for iron content, JL 3, PL 406, and L 4147 for zinc content, and L 4596, PL7, and L 4147 for PA content (**Figure 2**). The pattern of mineral (Fe & Zn) and PA content distribution among genotypes and locations was depicted using a box plot (**Figure 3**).

### Character association analysis

The association between test locations and seed Fe, Zn, and PA was investigated using Spearman’s correlation analysis (**Supplementary Figure 1**). When it came to seed Fe concentration, it was revealed that Delhi and Kanpur showed a positive significant association. When it related to seed zinc concentration, Kanpur and Sagar showed a negative correlation, but when it got to PA, all six places had a positive correlation. According to the significant correlation between Sehore and Sagar, the examined genotypes had a lot in common when it comes to seed iron and zinc levels. Spearman’s correlation analysis was used to explore the relationship between seed iron, zinc, and PA. Seed Fe and seed Zinc levels were found to have a significant positive relationship (**Supplementary Figure 2**). Both attributes can be increased as a result of this relationship.

### Evaluation of genotypes

**Figure 4:**
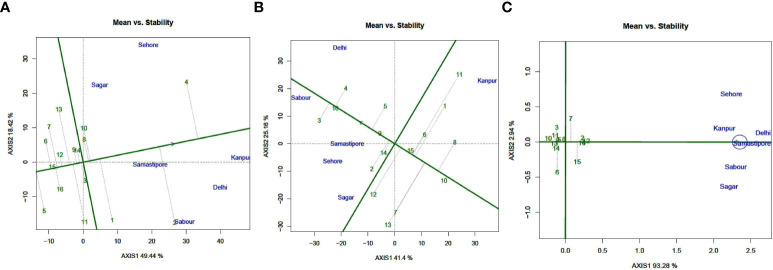
ean vs. Stability view of the GGE biplot of 16 lentil genotypes across 6 testing locations. **(A)** Fe, **(B)** Zn, and **(C)** PA). There was no transformation of data (transform = 0), and data were centered by means of the environments (centring = 2). The biplot was based on “row metric preserving.” Numbers correspond to genotypes as listed in **Table 1**.

Using a “AEC” perspective of the biplot, the genotype’s average performance and consistency across places were graphically represented (**Figure 4**). The single arrow-head line on the graph known as “AEC abscissa,” which crosses through biplot origin, indicates more seed iron. Seed iron concentration was higher in L 4147 (Pusa Vaibhav) (4), L4596 (2), DPL 62 (Sheri) (1), and K 75 (Mallika) (10) types, as shown in **Figure 4A**. The length of a genotype’s projection in absolute terms is commonly used to determine genotypic stability. The genotypes with the highest stability, i.e., a projection on AEC close to zero, and the highest seed iron content (a bigger negative projection on AEC) would be the best performers. As a result, the most “ideal” genotype was identified to be L 4076 (Pusa Shivalik) (8), with short projection from the “AEC abscissa” and optimal Iron levels. Genotypes that are more “desirable” are those that are closer to the “ideal” genotype. As a result, K-75 (10) and DPL 62(Sheri) (1) were designated as “desirable” genotypes because they were closer to the “ideal” genotype, with optimal iron and consistent performance.

The seed zinc concentration is higher in L4717 (Pusa Ageti) (14), L4596 (2), BM 4(9), DPL 58 (5), and PL 639 (16) (**Figure 4B**). AEC close to zero rated L4717 (Pusa Ageti) the “ideal genotype” for seed zinc concentration. L4596 (2), BM-4, and DPL 58 (5) were classified “ideal” genotypes because they were close to the “ideal” genotype, with optimum seed zinc concentration and consistent performance.

**Figure 4C** shows that seed phytic acid concentrations were higher in IPL 406 (15), PL639 (16), L4596 (2), and PL 7 (12). AEC found WBL 77 to be the “ideal genotype” for seed PA content because it was close to zero. IPL 406 (15), PL639 (16), L4596 (2), and PL 7 (12) were classified “ideal” genotypes because they were close to the “ideal” genotype, with optimum seed PA content and consistent performance.

### Evaluation of the environments

**Figure 5:**
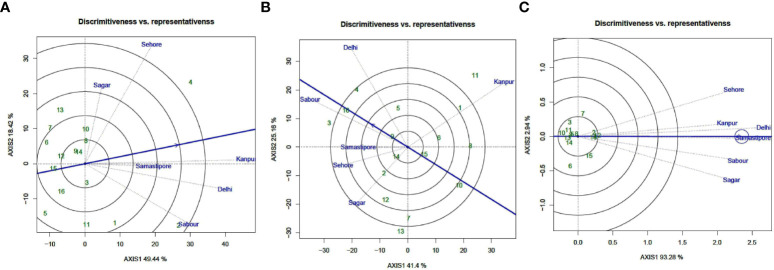
“Discrimitiveness vs. Representativeness” view of test locations based on GGE biplot of 16 lentil genotypes across 6 testing locations. **(A)** (Fe), **(B)** (Zn), and **(C)** (PA). There was no transformation of data (transform = 0), and data were centered by means of the environments (centring = 2). The biplot was based on “row metric preserving.” Numbers correspond to genotypes as listed in **Table 1**.

Among the test locations for seed iron concentration, Kanpur had the longest environmental vector, followed by Sehore, Delhi, and Sabour, with Samastipur having the shortest projection (**Figure 5A**). As a result, Kanpur was chosen as having the most “discriminating locations” with potential for genetic discrimination.

Delhi had the longest environmental vector among the test locations for seed zinc concentration throughout the year (2018-19), followed by Kanpur and Sabour, and Samastipur had the shortest projection (**Figure 5B**). As a result, in terms of genotype discrimination power, Delhi was categorized as one of the most “discriminating environments.”

During the year (2018-19), Delhi had the longest environmental vector for seed PA content, followed by Samastipur and Sehore, with Kanpur having the shortest projection (**Figure 5C**). Delhi was thus designated as one of the most “discriminating locations” in terms of genotype discrimination power. The solitary arrow-head line in the graph is labelled “AEC abscissa.” The stronger the “representative” power of the place, the smaller the angle between the environment vectors and the “AEC abscissa.”

Kanpur, followed by Samstipur, had the shortest angle with the AEC during the testing year, and were thus chosen as the most “Representative” test locations for seed iron concentration, whereas Sabour and Delhi were chosen as the most “Representative” test locations for seed zinc content. Samastipur was found to be the most “Representative” test location for seed PA content, followed by Delhi. Locations with high “discrimination” power but low “representativeness,” such as Sehore and Sagar, should be investigated for finding stable genotypes for seed iron and zinc content.

### Mega environments

**Figure 6:**
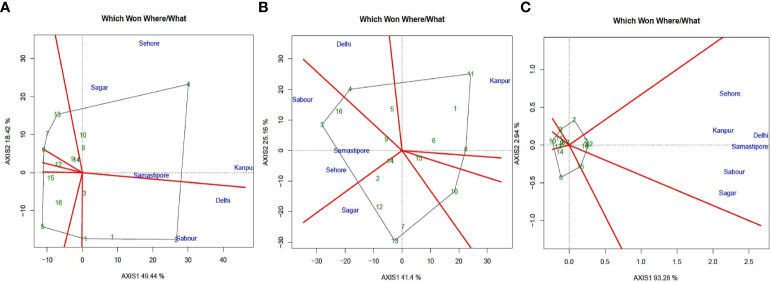
“Which-won-where” view of the GGE biplot of 16 lentil genotypes across 6 testing locations. **(A)** (Fe), **(B)** (Zn), and **(C)** (PA). There was no transformation of data (transform = 0), and data were centered by means of the environments (centring = 2). The biplot was based on “row metric preserving.” Numbers correspond to genotypes as listed in **Table 1**. Locations are: A, Delhi; B, Kanpur; C, Sehore; D, Sagar; E, Sabour and F, Samstipore.

GGE biplot employs a two-dimensional polygon visualization in the form of a “which won-where” polygon to detect genotypes for a certain production environment. Perpendicular lines were drawn from the biplot’s origin to each side of the polygon to partition the biplot into numerous sectors, with one “winning” genotype placed at the polygon’s vertex for each sector. L4147 (Pusa Vaibhav) (4) was revealed to have a substantially greater iron concentration and to be far from the origin, indicating that the performance was constant (**Figure 6A**). PL 6 (13), L4596 (2), L4717 (Pusa Ageti) (14), JL 3 (6), and WBL 77 (7) also had significant seed iron concentrations. DPL 58 (5), on the other hand, was identified downstream from the origin, exactly opposite L4147 (Pusa Vaibhav) (4), and was thus identified as the genotype with the lowest seed iron concentration. L4717(Pusa Ageti) (14) exhibited the most consistent performance of all the genotypes with moderate to medium iron content when placed near to the “AEC abscissa” with the least projection onto the “AEC ordinate.” The equality lines divided the plot into seven pieces. These sectors could be labelled “Mega Environment,” meaning that there is environmental unpredictability and G x E interaction.

VL 520 (11) had the highest mean zinc content and was far from the origin, indicating that its performance was consistent, according to this analysis (**Figure 6B**). Seed zinc content was also high in L 4076 (8), K 75 (Mallika) (8), IPL 321 (3), and L 4147 (Pusa Vaibhav) (4). PL 6 (13) was discovered downstream from the origin, right across from VL 520 (11), and was thus recognized as the low seed zinc content genotype. Sagar, Sehore, Samastipur, and Kanpur were single “mega environments” with different ecological features and genotypic reactions to seed iron concentration. The two “mega-environments” were located in Delhi and Sabour. The concentration of zinc in seeds was divided into four different “mega habitats.” Kanpur is part of a single “mega environment,” while Sabour, Samastipur, and Sehore are out of their own. The third and fourth “mega environments,” respectively, are Delhi and Sagar. All of the locations were grouped into a single habitat for phytic acid content, showing that the feature was less varied.

### GGE biplots by site regression (SREG) analysis

**Figure 7:**
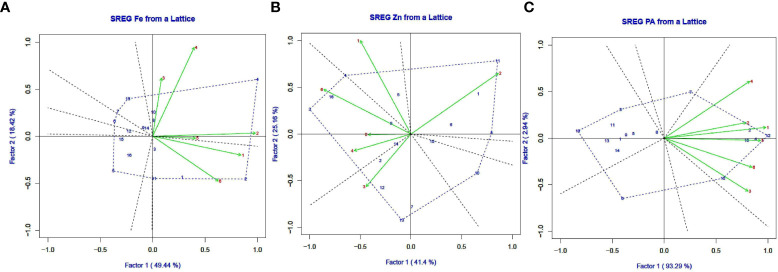
GGE biplots generated using SREG as an indication of seed iron content **(A)**, seed zinc content **(B)** and seed PA content **(A)** (Fe), **(B)** (Zn), and **(C)** (PA). for stability in lentil genotypes grown in six environments in India during 2018-2019. Genotype (G) and environment (E) codes are given in **Tables 1** and **2**, respectively.

**Figures 7A–C** show GGE biplots for seed iron, zinc, and PA content obtained by the SREG model. The discriminating power of the sites was determined by their proximity to the origin of the vertices between PC1 and PC2, and the scores of the cultivars furthest from the origin were joined to construct a polygon. The polygon encompassed all other genotypes, indicating which cultivars were the most stable based on their correlation with site scores. The genotypes that made up the polygon were the most responsive to their environment and were reflective of the greatest or worst performance. The L 4076 and K75 for Fe, JL 3 and DPL 62 for Zn, and WBL 77 for PA were stable as they were in polygon of GGE and their values were close to zero on the Y-axis. Concentric circles rippling around the average environmental coordinate (AEC) of a genotype focused GGE biplots encompass genotypes that are relatively similar in their overall desirability. Based on this criterion L4147 (Pusa Vaibhav) for seed iron content, IPL 321, L 4147 and PL 639 for seed zinc content and all genotypes for PA were under the desirable genotypes for wider adaptation.

### Genotype ranking based on their mean performance and stability

**Figure 8:**
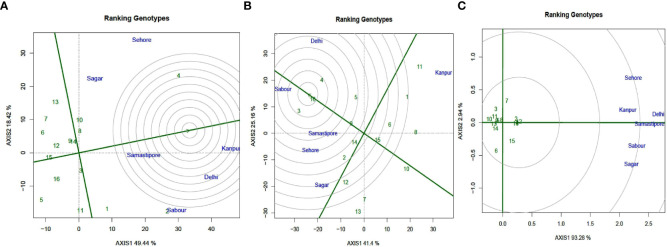
GGE-biplot based on genotype-focused scaling for comparison of the genotypes with the ideal genotype for **(A)** (Fe), **(B)** (Zn), and **(C)** (PA). Green and violet numbers stand for Genotypes and environments, respectively.

Using the average environment coordinate, ranking biplots were utilized to rate the genotypes according to their performance and stability (AEC). In the ranking biplot, an average environment axis (AEA) depicted by a single arrowhead line passing through the origin indicates that a genotype’s mean performance is superior. The ranking biplot AEC revealed that genotypes L 4147 (4), L4076 (8), IPL 321 (3), and DPL 62(1) had high mean Fe content and genotypes DPL 58 (5), BM-4 (9) and Pl 639 (16) had high mean Zn content in this study. Genotypes DPL 58 (5) and PL 7 (12) exhibited the lowest Fe and Zn levels, respectively (**Figure 8**). In PA, the majority of genotypes were close to the AEC, but WBL 77 (7) and JL 3 (6) were far away. The length of the vector between the genotype positions and the AEA in ranking biplot was used to assess genotype stability. Genotypes that are remote from the origin but on the AEA or near to it have the best performance and stability. As a result, L 4147 was the most stable genotype for Fe, while IPL 321(3), L 4147(4), and PL639 were the most stable genotypes for PA, with a high mean and shorter vector from AEA.

## Discussion

### Substantial genetic variations for grain mineral concentration

Lentil grains typically contain higher Fe and Zn attributable to breeding since genotypes have substantial genetic variation. According to other investigations (Thavarajah et al., 2009; Thavarajah et al., 2011; Karakoy et al., 2012; Sen Gupta et al., 2013), the lentil gene pool comprises a wide range of genetic diversity for these micronutrients. Iron levels in red and green lentil genotypes ranged from 43 to 132 mg/kg, while zinc levels ranged from 22 to 78 mg/kg, according to an investigation of 1,600 genotypes of lentils (Sarker et al., 2007). In a multi-location, multi-year experiment in Saskatoon, Canada (Thavarajah et al., 2009), significant genetic diversity was found in 19 lentil genotypes for grain iron (73-90 mg/kg) and zinc (44-54 mg/kg) levels. In 1,000 core collection common bean germplasm at the CIAT (International Center for Tropical Agriculture), the levels of zinc (21 to 54 mg/kg) and iron (34 to 89 mg/kg) demonstrated a wide range of variability (Welch & Graham, 2002; Welch, 2002).

The Fe, Zn, and phytic acid contents between lentil cultivars were clearly distinguishable, according to the current study. Across the study locations, Fe and Zn levels varied from 114.10 to 49.90 mg/kg and 74.62 to 21.90 mg/kg, respectively. The range of phytic acid concentrations in present investigations ranged 0.76 to 2.84 g/100g (dw) (**Table 4**). While, lentils had phytic acid contents of 0.40 to 1.29 g/100 g, 0.43 to 0.77 g/100 g in pea (*Pisum sativum*), and 1.17 to 1.70 g/100 g in soybean (*Glycine max*) (Vojtíšková et al., 2010), chickpeas 0.28–1.60 g/100 g, kidney beans 0.61–2.38 g/100 g, (Lehrfeld, 1994) and peanuts 0.17–4.47 g/100g (Venkatachalam and Sathe, 2006). Cereal crops, such as maize germ (6.39 g/100g), wheat bran (2.1–7.3 g/100g) (Harland et al., 1986), and rice bran (2.56–8.7 g/100g) (Kasim and Edwards, 1998), have a much higher range than pulses. Plant breeding and molecular approaches have convincingly demonstrated that lentil crops can be used as bio-fortified crops Thavarajah et al., 2011).

**Table 4:** Mean of seed iron, zinc and phytic acid in 16 genotypes of lentil at six locations during 2018–2019.

S.No	Delhi			Kanpur			Sagar			Sehore			Samastipore			Sabour			Mean		
	Fe	Zn	PA	Fe	Zn	PA	Fe	Zn	PA	Fe	Zn	PA	Fe	Zn	PA	Fe	Zn	PA	Fe	Zn	PA
1	82.14	54.70	1.08	80.73	53.42	1.41	63.55	35.56	1.47	65.26	46.19	1.48	70.38	34.96	1.27	98.21	46.81	1.23	76.71	45.27	1.33
2	114.10	47.62	2.49	80.01	36.66	2.52	76.57	66.29	2.65	66.77	42.31	2.82	97.02	48.70	2.47	105.10	64.88	2.42	89.93	51.08	2.56
3	87.43	66.35	1.03	66.32	21.90	1.83	75.43	51.57	1.31	60.93	53.45	1.61	87.46	52.19	1.18	72.88	74.62	1.06	75.07	53.35	1.34
4	96.72	70.29	2.57	96.30	53.93	2.79	87.71	64.24	2.62	100.29	60.91	2.61	94.19	64.15	2.57	94.46	68.17	2.54	94.94	63.61	2.62
5	64.68	66.76	1.25	57.09	38.29	1.49	72.00	48.42	1.69	53.90	48.40	1.75	75.21	35.91	1.25	85.79	57.58	1.29	68.11	49.23	1.45
6	65.80	53.17	0.94	56.89	52.60	1.49	73.42	53.91	2.11	71.65	45.61	1.21	78.35	43.49	0.94	72.28	47.57	1.47	69.73	49.39	1.36
7	62.89	42.44	1.96	50.61	32.53	1.96	86.37	64.98	1.96	75.11	55.78	2.64	76.10	32.46	1.79	86.78	47.26	1.67	72.98	45.91	2.00
8	82.96	47.42	1.52	77.98	47.43	1.79	75.43	46.78	1.82	71.38	25.65	1.89	64.69	38.02	1.52	68.06	44.18	1.52	73.42	41.58	1.68
9	89.80	67.73	1.15	55.48	34.73	1.78	86.51	52.69	1.45	65.27	52.43	1.42	83.24	52.81	1.26	72.46	43.86	1.25	75.46	50.71	1.39
10	82.66	38.94	0.76	68.21	47.15	0.98	79.71	49.32	0.95	73.95	45.78	1.13	82.02	42.21	0.76	69.36	39.88	0.93	75.98	43.88	0.92
11	83.40	58.32	0.92	60.11	63.92	1.79	74.11	38.15	1.24	55.68	32.65	1.30	86.90	40.03	1.30	91.46	50.01	0.98	75.28	47.18	1.26
12	81.54	44.92	2.71	51.39	25.16	2.71	69.93	54.50	2.71	73.70	53.40	2.84	78.46	45.55	2.71	74.46	54.51	2.71	71.58	46.34	2.73
13	80.23	38.96	1.06	49.90	30.18	1.06	94.13	63.68	1.28	74.35	50.73	1.35	74.96	57.39	1.18	75.48	43.81	1.22	74.84	47.46	1.19
14	79.15	49.23	1.16	55.46	35.26	1.16	76.32	51.68	1.43	74.65	46.43	1.34	88.48	44.13	1.34	82.53	61.61	1.31	76.10	48.06	1.29
15	75.02	46.69	2.20	52.40	46.25	2.23	69.47	49.44	2.61	68.14	41.20	2.01	82.71	64.72	2.55	74.11	49.54	2.20	70.31	49.64	2.30
16	86.33	68.24	2.46	57.86	24.25	2.68	67.76	49.61	2.55	60.69	47.07	2.52	75.45	45.97	2.54	69.05	72.99	2.43	69.53	51.35	2.53
Mean	82.18	53.86	1.58	63.55	40.23	1.85	76.78	52.55	1.87	69.48	46.75	1.87	80.98	46.42	1.66	80.78	54.21	1.64	75.62	49.00	1.75
Variance	156.45	121.39	0.48	184.21	150.80	0.35	67.84	79.09	0.36	113.71	74.62	0.38	72.61	95.30	0.45	133.79	123.47	0.37	51.01	24.28	0.37
SD(E)	3.13	2.75	0.17	3.39	3.07	0.15	2.06	2.22	0.15	2.67	2.16	0.15	2.13	2.44	0.17	2.89	2.78	0.15	1.79	1.23	0.15
SD(D)	12.51	11.02	0.69	13.57	12.28	0.59	8.24	8.89	0.60	10.66	8.64	0.62	8.52	9.76	0.67	11.57	11.11	0.61	7.14	4.93	0.61
CV	15.22	20.46	43.89	21.36	30.53	31.87	10.73	16.92	32.23	15.35	18.48	33.03	10.52	21.03	40.13	14.32	20.50	37.10	9.44	10.05	34.73

### Genotype, genotype × environment interaction

The levels of seed iron and zinc in nineteen lentil lines produced over a two-year period in eight locations in Saskatchewan, Canada, demonstrated that genotype x location interactions had a significant effect primarily on zinc content, but not on iron content (Thavarajah et al., 2009). In wheat, genotype x location interactions were found to be significant for Zn and Fe levels in both wild and modified cultivars (Gomez-Becerra et al., 2010). In the instance of durum wheat, 46 genotypes were tested for Fe, Zn, and phytic acid content in two habitats, and the genotype, environment, and their interaction revealed highly significant impacts (G x E). The effect of the environment was particularly strong in the case of phytic acid: Fe ratio and phytic acid (Magallanes-López et al., 2017). The genotype, environment, and genotype x environment interactions for iron, zinc, and phytic acid concentration were also quite significant in the current analysis (**Tables 3**), implying that the environmental conditions as stated in **Table 2** are important. Micronutrient mobility from root to seed is likely to be influenced by growth seasons, in addition to soil conditions. These data reveal that crop mineral properties are influenced by both heredity and environmental factors. To improve mineral content through breeding, it is vital to examine the location of certain environmental circumstances as well as the genetic make-up of the genotype.

The current study aimed to shed light on the impact of environmental and genotype-by-environment interactions on the responsiveness of lentil genotypes to nutritional and anti-nutritional attributes. The incoherent response of genotypes and locations across sites revealed the impact of the environment on the volatility of the parameters investigated. Quantitative traits control the traits under consideration. Many genes with lesser, comparable, and cumulative effects influence the expression of quantitative features. The genotypes, as well as the interaction of all of these variables with genotypes, contributed to superior/inferior performance and genotype stability across sites. The genotype with lesser effect of G X E interactions performs stable way in the expression of the traits. These mineral-rich stable cultivars grown in the ideal environment would not only increase lentil output but also productivity among marginal and small farmers. Furthermore, a large number of genotypes, many sites, and multiple years will produce robust results. In addition to the aforementioned points, *in vivo* and *in vitro* studies in lentils must be conducted so that the benefit of the high Fe method can be substantiated as one of the best approaches in biofortification initiatives.

### Role of broad sense heritability (H^2^) in mineral bio-fortification

Heritability is critical in the genetic improvement of quantitatively inherited characteristics through selection. Estimates of trait heritability distinguish the amount of total phenotypic variance caused by genotypes and environmental factors, and they tell us how much response may be obtained by selecting any plant population over the initial genetic pool (Lynch and Walsh, 1998). Understanding trait heredity is crucial for plant breeders. In our study, the broad sense heritability (H^2^) for Fe (0.94 to 0.99) and Zn (0.53 to 0.99) content was moderate to high, indicating that genotypic impacts across contexts accounted for a large percentage of the variability in the character (**Table 5**). However, strong estimates of broad sense heritability (H^2^) were found in phytic acid levels across regions (0.96 to 0.99 percent). The genotypes analyzed have genetic potential, as evidenced by moderately high to high levels of heritability in our tested samples. As a result, it can be utilized to develop lentil cultivars with increased iron and zinc content while lowering phytic acid levels. Despite the fact that both genotype and genotype x environment interactions accounted for a significant amount of overall phenotypic variance, a sufficient fraction of genetic variability is proven to be heritable.

**Table 5:** Calculations heritability estimates based on the geography for grain iron (Fe), zinc (Zn), and phytic acid (PA).

S.No	Environment	Board sense heritability		
		Fe	Zn	PA
1	Delhi	0.9824	0.9912	0.9939
2	Kanpur	0.9902	0.9925	0.9770
3	Sabour	0.9695	0.5318	0.9819
4	Sagar	0.9855	0.9884	0.9868
5	Samastipore	0.9481	0.9897	0.9948
6	Sehore	0.9874	0.9901	0.9600
	**Mean**	**0.9772**	**0.9140**	**0.9824**

The bold values are the mean values of the respective traits.

In lentils, previous research found moderate heredity for Fe concentration, but low to relatively high heritability for Zn content (Thavarajah et al., 2009; Kumar et al., 2018). Very high estimates of broad-sense heritability (h^2^bs) for Fe and Zn concentration were found in black gram (*Vigna mungo* (L) Hepper), whereas lower heredity in phytic acid indicated a substantial environmental influence (Singh J. et al., 2017). Broad-sense heritability (h^2^bs) for both grain Fe and Zn ranged from moderate to high values in pearl millet (Velu et al., 2007; Gupta et al., 2009).

### Grain iron and zinc can be increased simultaneously

Positive trait relationships encourage breeders to simultaneous improvement of two or more traits. Our findings demonstrated that iron and zinc levels had a strong and favorable association. However, both minerals had a favorable but non-significant relationship with phytic acid (**Figure 6**). The concentration of iron revealed a non-significant and positive connection with the content of zinc in the lentil studies mentioned before (Karakoy et al., 2012; Kumar et al., 2018). Fe content in black gram was shown to have a high positive correlation with Zn content. There was a clear association between phytic acid concentration and the minerals (Fe and Zn) (Singh et al., 2017a). Rice (Kabir et al., 2003; Inabangan-Asilo et al., 2019), wheat (Gomez-Becerra et al., 2010), maize (Long et al., 2004; Mallikarjuna et al., 2015), pearl millet (Pucher et al., 2014), and sorghum (Reddy et al., 2005) have all been found to exhibit positive relationships between these two minerals. These findings show that simultaneous selection for high iron and zinc levels in particular crops is possible. Correlations between seed iron sites in Delhi and Kanpur, Sehore and Sagar were positive and significant. In terms of seed zinc concentration, there was a substantial negative association between Kanpur and Sagar, but a large positive correlation between Sagar and Sehore. Grain mineral micronutrients (Zn and Fe) between two locations were highly and positively associated in milled and brown rice (Bollinedi et al., 2020).

Genetic improvement has previously increased the concentration of iron and zinc in crops such as wheat, rice, and common bean (Welch and Graham, 2002; Welch, 2002). Our findings revealed a wide range of Fe and Zn content genetic variability that can be leveraged to create nutritionally dense Fe and Zn lentil cultivars. It may thus be a feasible method for treating micronutrient deficiency in human beings who consume lentils on a daily basis. The frequency of the association varies based on the situation. These findings imply that both genetic and environmental factors influence mineral association. Mineral association in grain may have a genetic the basis due to mineral transporter genes co-segregating in genotypes and/or the availability of common transporters for many minerals (Schaaf et al., 2005).

### Ideal and desirable genotypes

Plant breeders tend to identify genotypes that have the least interacting influence with a broad adaptation environment in their extensive plant breeding program. Multi-environmental studies (Kang, 2002) can uncover minor geographical characteristics with consistent performance across sites, as well as small temporal variables with consistency over years. In the GGE biplot’s “Mean vs. Stability” view, the “AEC ordinates” show a larger GE interaction effect in both directions and poor stability (Yan and Tinker, 2006), whereas the vector projection of the genotype to the “AEC abscissa” indicates mean performance (Yan & Falk, 2002) [34]. In addition, in the current study, K-75 (10), as well as DPL 62 (Sheri) (1), were identified as “desirable” genotypes, and were found to be closer to the ideal genotype, L4076 (Pusa Shivalik) (8). For Zn concentration, L4717 (Pusa Ageti) was deemed the “ideal genotype,” whereas L4596 (2), BM-4, and DPL 58 (5) were deemed “desirable.” For phytic acid content, WBL 77 was deemed “ideal,” while IPL 406 (15), PL639 (16), L4596 (2), and PL 7 (12) were deemed “desirable.” Those “ideal” genotypes had more mineral content, indicating robust stability (Yan et al., 2007), with higher negative projection on the ATC abscissa and less projection on AEC ordinates. These techniques have been used to successfully identify stable genotypes in chickpea (Erdemci, 2018; Misra et al., 2020), mungbean (Ullah et al., 2011), lentil (Darai et al., 2020), soybean (Mwiinga, 2018), faba bean (Fikere et al., 2008; Tekalign et al., 2017), and maize (Beyene et al., 2011; Tonk et al., 2011). The “mega environment” can be successfully depicted using GGE biplot methods in a “which-won-where” approach (Gauch and Zobel, 1997; Yan and Kang, 2002; Yan et al., 2007). The goal of mega-environment identification is to grasp the region’s complicated GEI pattern in order to exploit specific adaptability and increase selection response (Yan et al., 2011). Earlier studies defined a “mega environment” as a collection of places with consistent genotypic responses (Yan et al., 2000; Yan and Rajcan, 2002; Yan & Tinker, 2006). Many investigations, including lentil (Singh et al., 2017a; Jeberson et al., 2019), chickpea (Erdemci, 2018), uradbean (Gupta et al., 2020b), mungbean (Asfaw et al., 2012), pea(Rana et al., 2020), pigeonpea (Kumar et al., 2021), and soybean (El-Harty et al., 2018), used these methodologies to depict mega environments.

In the current study, the genotypic response to grain minerals and phytic acid content was shown to be identical in each “mega environment” tested. It is critical to control the synchrony of study locations and convergent breeding activities in a location-specific manner in order to improve the precision of lentil bio-fortification. The goal of this study was to find out more about how environmental and genotype-by-genotype interactions influence lentil genotype responses to grain mineral and phytic acid concentrations.

### Lentil as bio-fortification tool

The inconsistent response of genotypes and locations to the environmental influence on mineral and phytic acid content reflected the environmental effect on mineral and phytic acid content. “Ideal” and “desirable” genotypes for grain iron and zinc content were successfully discriminated against in our study. Not only the stable cultivars like K-75 (for Fe) and L4596 (for Zn) but also “desirable” genotypes with consistent performance like L4076 (Pusa Shivalik) (for Fe) and L4717 (Pusa Ageti) (for Zn) genotypes were recommended for use. In terms of determining the levels of phytic acid content and its stability across locations, our study adds to the current knowledge. The decreased inhibitor concentration, such as phytic acid, will improve the bioavailability of grain minerals in legumes. Furthermore, genetic variation in iron and zinc concentrations can aid in the identification of genes/quantitative trait loci (QTL) linked to iron and zinc consumption and accumulation. Furthermore, a genetic examination of iron and zinc levels in seeds demonstrated the impact of environmental variables. Thus, location testing or region-specific breeding can aid in the generation of lentil varieties that are high in iron and zinc.

The most prevalent problem among Asian and African women and preschool children is iron and zinc deficiency. For males and women, the RDA for iron is 8 mg/day and 18 mg/day, respectively, whereas the RDA for zinc is 11 mg/day for men and 8 mg/day for women aged 19 and up (https:/ods.od.nih.gov/professional/factsheets/IronHealth). The lentil genotypes studied were able to deliver a significant amount of RDA for Iron and Zinc in our study. For example, genotypes of L4147 (Pusa Vaibhav) had the highest average Fe content in their seeds (94.94 mg/kg), which could be enough to supply 168.91 and 136.24 percent of RDA Fe intake for adult males and females, respectively. The same line L4147 (Pusa Vaibhav) contained 63.61 mg/kg of seed Zn, which is sufficient to give 141.02 and 155.31 percent of RDA (in case of Zn) for adult men and females, respectively.

The iron-rich nature of lentil variety L4147 (Pusa Vaibhav) has been established by numerous earlier investigations (Kumar et al., 2014 and Kumar et al., 2019). Our findings also show that suitable and stable mineral-rich lentils like L 4076 and L4717 can be used as donors for further mapping and molecular analysis. The decreased phytic acid content of these discovered types naturally increases the bioavailability of grain micronutrients in poor people’s diets. These cultivars are critical trait donors for future mapping and tagging investigations. They have the added benefit of being able to directly release or notify other zones, improving lentil yield and productivity, because they are newly released cultivars. Transcriptomics studies using these lines could provide insight into the paths for grain mineral absorption, transport, and storage in lentils and other pulses. The mapping populations developed through these parents make it much easier to find the genes and QTLs involved in grain mineral uptake, transportation, and regulation. The proposed trait-specific desirable genotypes, as well as large environments like Sagar and Sehore, will revolutionize lentil cultivation by enhancing productivity and production. Specific labeling and marketing methods must be developed in order to popularize bio-fortified crops. Direct production will be profitable, and immediate inclusion in the normal diet through the public distribution system (PDS) will boost micronutrient consumption in poor families, reducing micronutrient deficiency. In order to address the issue of hidden hunger, investigations on the bioavailability of these plant-based Fe and Zn must be investigated.

## Conclusions

- The grain iron (Fe), grain zinc (Zn), and grain phytic acid(PA) concentrations in commercially cultivated lentil genotypes showed significant genetic variations in different locations.
- The environment (E) and G x E (Genotype x Environment interactions) had an impact on the concentration of grain Fe, Zn, and phytic acid (PA).
- Our research identified strong positive correlation between the contents of Fe and Zn, a strategy for simultaneously increasing Fe and Zn in lentils may be recommended.
- In addition, our study found that the stable and ideal lentil varieties L4076 (Pusa Shivalik) for Fe concentration and L4717 (Pusa Ageti) for Zn content, with lower phytic acid contents, will not only play a crucial role as stable donors in lentil bio-fortification but will also enable the expansion of bio-fortified crops to achieve health and nutrition security.
- The lentil genotypes identified in our study were able to deliver a significant amount of Recommended Dietary Allowance (RDA) for Iron and Zinc.
- In case of genotypes of L4147 (Pusa Vaibhav) had the highest average Fe content in their seeds (94.94 mg/kg), which could be enough to supply 168.91 and 136.24 percent of RDA Fe intake for adult males and females, respectively.
- The same line L4147 (Pusa Vaibhav) contained 63.61 mg/kg of seed Zn, which is sufficient to give 141.02 and 155.31 percent of RDA (in case of Zn) for adult men and females, respectively.
- The ideal and stable mineral-rich lentils such as L 4076 and L4717 can serve as donors for further mapping and molecular dissection.
- Direct production of L 4147 (Pusa Vaibhav) and L 4717 not only profitable, but direct inclusion in the normal diet through the public distribution system (PDS) will boost micronutrient consumption in poor families, reducing micronutrient deficiency.
- Furthermore, a large number of genotypes, more umber of environments, and many years will yield reliable data. In addition to this, *in vivo* and *in vitro* studies in lentils are required to validate the high Fe method as one of the best approaches in biofortification initiatives.

## Data availability statement

The original contributions presented in the study are included in the article/**Supplementary Material**. Further inquiries can be directed to the corresponding authors.

## Author contributions

MA, HD, GM, PY, and JT planned and designed the research. MA, TD, AK, VK, AP, and AS performed the experiment. VR, P, TB, KT, and PG helped in data recording. MA, NR, RB, AK, SG, JK, and AP prepared the manuscript. MA, SK, PY, and VK edited the manuscript for publication. All authors contributed to the article and approved the submitted version.

## Supplementary material

Supplementary Figure 1 Pearson’s correlation between six test locations for seed Fe, Zn, and phytic acid during 2018-19. _P < 0.05 are boxed. Locations are: A: Delhi, B Kanpur; C: Sehore; D: Sagar; E: Sabour and F: Samstipore. (p <0.05 are boxed).

Supplementary Figure 2 Pearson’s correlation between seed Fe, Zn, and phytic acid A: Iron (Fe); B: Zinc (Zn), C: Phytic Acid (PA) (p <0.05 are boxed).
