# Supplementary material for: Strategies for identifying stable lentil cultivars (Lens culinaris Medik) for combating hidden hunger, malnourishment, and climate variability
Source: Front Plant Sci. 2023 Jul 13;14:1102879. doi: 10.3389/fpls.2023.1102879 (PMC10374012; doi:10.3389/fpls.2023.1102879)
Supplement: Supplementary file 1 [file Table_1.docx]

**Supplementary** Table 1. Soil analysis data of different locations

| Soil analysis data of different locations | Delhi | Kanpur | Sehore | Sagar | Sabour | Samastipore |
| --- | --- | --- | --- | --- | --- | --- |
| pH | 8.52 | 8.24 | 7.42 | 7.55 | 7.26 | 7.49 |
| ES (ds/m) | 0.38 | 0.37 | 0.22 | 0.23 | 0.42 | 0.38 |
| Organic Carbon Content | 0.44 | 0.41 | 0.54 | 0.34 | 0.51 | 0.46 |
| Available N (kg/ha) | 184 | 174 | 150 | 170 | 178 | 183 |
| Available P (kg/ha) | 25.5 | 26.2 | 13.5 | 14.5 | 28.24 | 26.57 |
| Available K (kg/ha) | 290 | 282 | 376 | 550 | 440 | 434 |
| Texture | Sandy loam | Sandy loam | Black cotton | Black cotton soil | loamy | loamy |
| Soil Fe concentration (mg/kg) | 5.01 | 5.23 | 4.5 | 4.2 | 4.58 | 5.31 |
| Soil Zn concentration (mg/kg) | 1.68 | 1.62 | 0.98 | 0.62 | 1.23 | 1.35 |
