# Supplementary figures and images for: Strategies for identifying stable lentil cultivars (Lens culinaris Medik) for combating hidden hunger, malnourishment, and climate variability
Source: Front Plant Sci. 2023 Jul 13;14:1102879. doi: 10.3389/fpls.2023.1102879 (PMC10374012; doi:10.3389/fpls.2023.1102879)

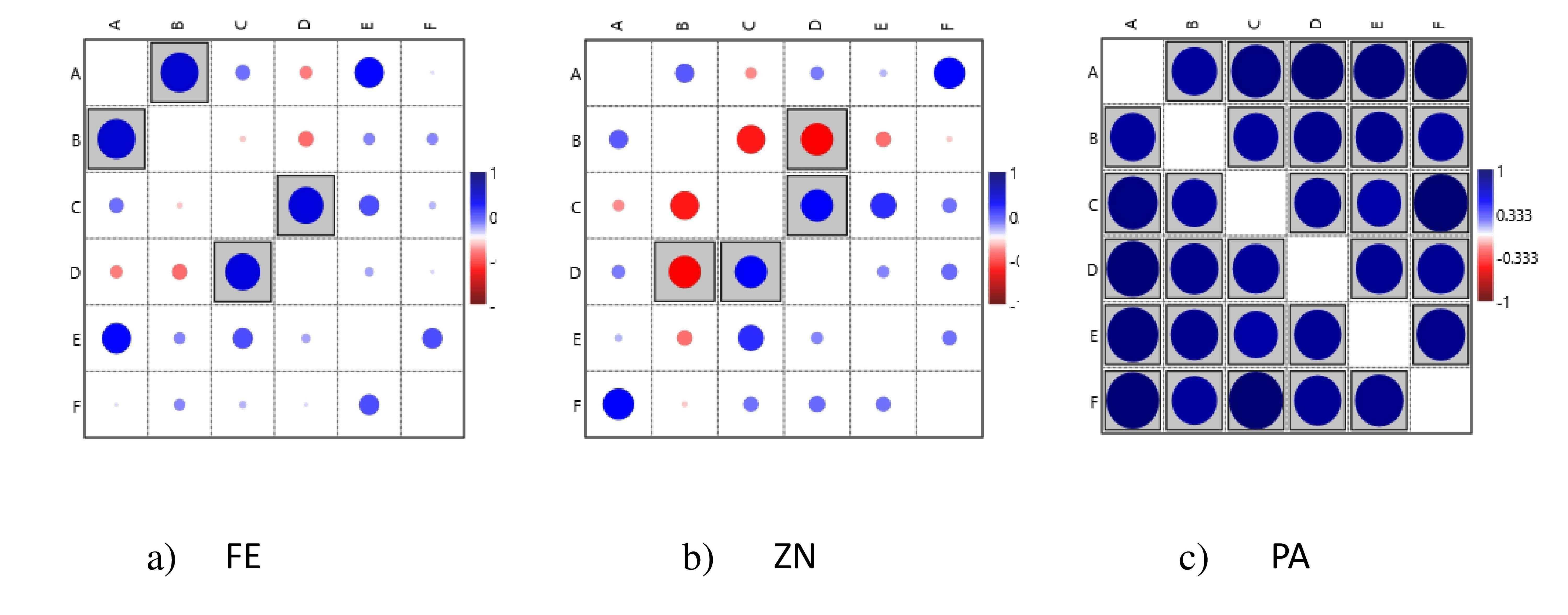

Supplement: Supplementary Figure 1 — Pearson’s correlation between six test locations for seed Fe, Zn, and phytic acid during 2018-19. _P < 0.05 are boxed. Locations are: A: Delhi, B Kanpur; C: Sehore; D: Sagar; E: Sabour and F: Samstipore. (p <0.05 are boxed). [file Image_1.jpg]

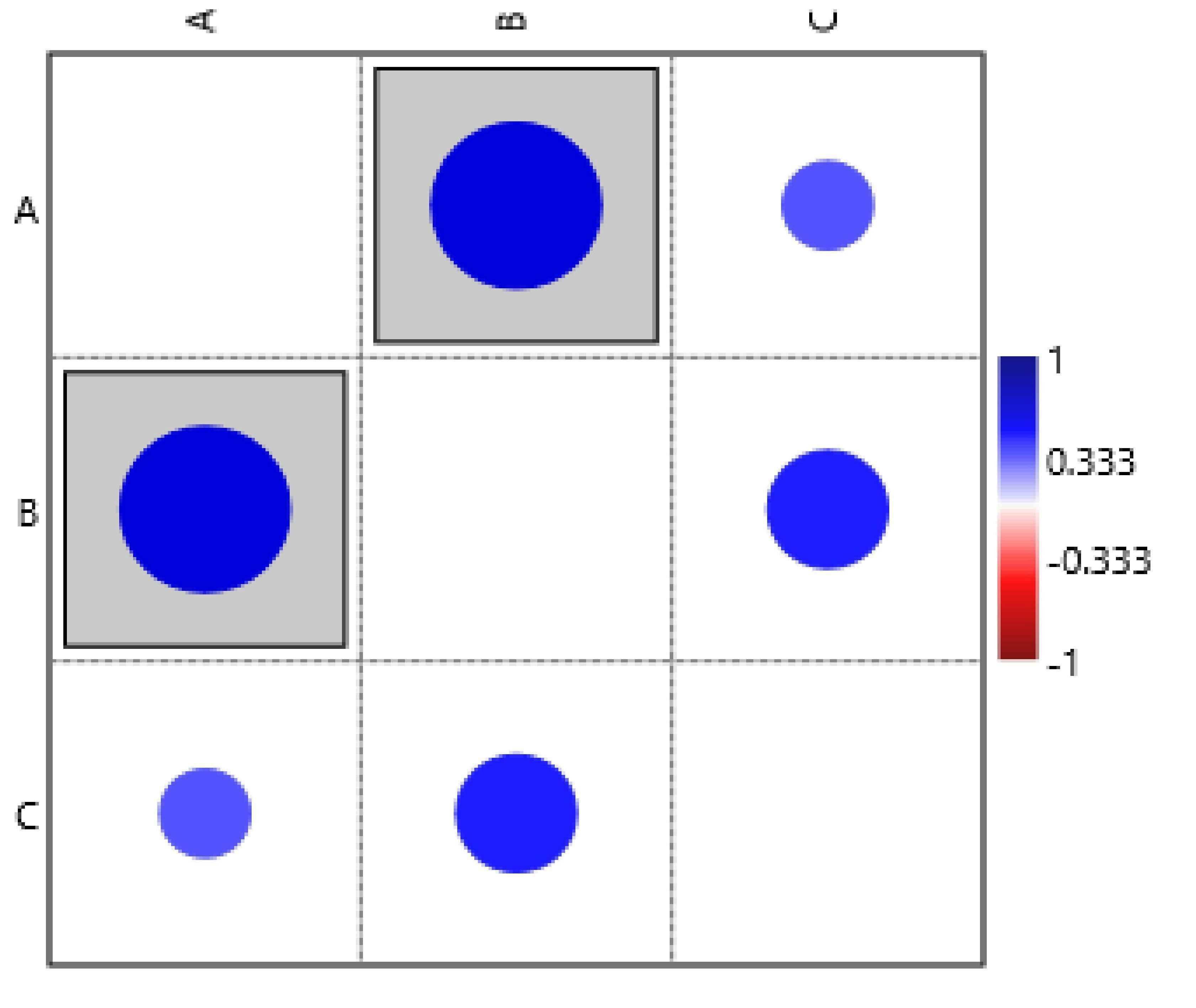

Supplement: Supplementary Figure 2 — Pearson’s correlation between seed Fe, Zn, and phytic acid A: Iron (Fe); B: Zinc (Zn), C: Phytic Acid (PA) (p <0.05 are boxed). [file Image_2.jpg]
